# The Value of ABCD2F Scoring System (ABCD2 Combined with Atrial Fibrillation) to Predict 90-Day Recurrent Brain Stroke

**DOI:** 10.1155/2016/8191659

**Published:** 2016-08-23

**Authors:** Mostafa Almasi, Nader Hodjati Firoozabadi, Faeze Ghasemi, Mojtaba Chardoli

**Affiliations:** ^1^Department of Neurology, Rasoul-e-Akram Hospital, Iran University of Medical Sciences, Tehran 14456 13131, Iran; ^2^Department of Emergency Medicine, Rasoul-e-Akram Hospital, Iran University of Medical Sciences, Tehran 14456 13131, Iran; ^3^Department of Emergency Medicine, Haft-e-Tir Hospital, Iran University of Medical Sciences, Tehran, Iran

## Abstract

*Background*. The ABCD2 score is now identified as a useful clinical prediction rule to determine the risk for stroke in the days following brain ischemic attacks.* Aim*. The present study aimed to introduce a new scoring system named “ABCD2F” and compare its value with the previous ABCD2 system to predict recurrent ischemic stroke within 90 days of the initial cerebrovascular accident (CVA).* Methods*. 138 consecutive patients with the final diagnosis of ischemic CVA or TIAs who referred to emergency ward of Rasoul-e-Akram general hospital in Tehran from September 2012 to December 2013 were eligible. By adding a new score in the presence of atrial fibrillation to ABCD2 system, the new scoring system as ABCD2F was introduced and the risk stratification was done again on this new system.* Results*. The area under the curve for ABCD2 was 0.434 and for ABCD2F it was 0.452 indicating low value of both systems for assessing recurrence of stroke within 90 days of primary event. Multivariable logistic regression analysis showed that none of the baseline factors could predict 90-day recurrent stroke.* Conclusion*. ABCD2 and/or atrial fibrillation are not good scoring candidates for assessing the risk of recurrent stroke within first 90 days.

## 1. Introduction

Cerebrovascular events still remain as one of the major leading causes of death and disability all over the world. Only in the United States, the annual incidence of these events has been estimated to be 780.000 strokes, 600.000 as the primary strokes and others as recurrent attacks [1]. The management of cerebrovascular disorders has imposed a high cost on healthcare management systems with an estimation of 65.5 billion dollars in the USA in 2008 [2]. Much of this cost has been related to prevention and management of recurrent stroke. Studies have given point estimation of 3-year stroke recurrence rates between 6% and 25% [3–5]. Incidence rates of first stroke recurrence were highest in the first year after index stroke with a risk of 8.0% ranging from 3.5% to 1.8% within the second, third, fourth, and fifth years [6]. Ever, some potential risk factors have been identified to predispose patients to recurrent brain stroke including residual extracranial and intracranial occlusive disease and multiple and crescendo TIAs and hypertension (for early stroke recurrence) and advanced age, hypertension, heart disease, and diabetes mellitus (for late stroke recurrence) [5, 7–9]. In this regard, the main principles for recurrence of brain stroke include evaluation for risk factors and evaluation for cause (arterial and cardiac abnormalities), as well as management of the risk factors through lifestyle modification, using proper medications and surgical or endovascular interventions, if required. The first step for prognostic and preventive management of recurrent brain stroke is risk stratification of this event. Various scoring systems have been employed to stratify the risk for this event so far. The ABCD2 score is now identified as a useful clinical prediction rule to determine the risk for stroke in the days following primary brain ischemic attacks, particularly after TIAs [10]. This score has been structured based on five parameters of age, clinical features, duration of TIA, blood pressure measure, and diabetes mellitus that scores of each item are added together to produce an overall result ranging between zero and seven [11]. The follow-up period for this score can be either between 0 and 7 days of the cerebrovascular event (acute phase) or between 8 and 90 days (subacute phase) [12]. According to the risk stratification rule of this scoring system, the risk for recurrent stroke within 2 to 7 days ranged from 1.0% to 1.2% in the low-risk group, from 4.1% to 5.9% in the moderate-risk group, and from 8.1% to 11.7% in the high-risk group [12]. The expected 90-day risks of stroke in these groups were 3.1%, 10%, and 17.4%, respectively [13]. Interestingly, the relevance of some other risk profiles such as atrial fibrillation with increased risk for recurrent brain stroke has been proposed [14, 15], and thus it seems that adding this factor to common scoring systems such as ABCD2 can increase its prognostic value for these patients. The present study aimed to add the presence of atrial fibrillation as a potential risk factor to ABCD2 system and to modify it as a new scoring system named “ABCD2F.” Then, the value of this new system was compared to the previous ABCD2 system to predict recurrent brain stroke within 90 days of the initial cerebrovascular accident.

## 2. Materials and Methods

In this study, 138 consecutive patients with the final diagnosis of ischemic cerebrovascular accidents (CVA) or TIAs who referred to emergency ward of Rasoul-e-Akram Hospital in Tehran, Iran, from September 2012 to December 2013 were eligible for the study. The patients with hemorrhagic CVA or those who died within 90 days of discharge from the hospital were excluded (Table 1). The primary information of the patients was retrospectively collected by reviewing the recorded hospital files and baseline characteristics, including demographics, medical history, clinical findings, and also laboratory and imaging parameters. With regard to assessing the patients' sequel, it was followed up by telephone in order to assess the reappearance of the symptoms of CVA or TIAs. Furthermore, if the patients referred to emergency ward of the hospital within 90 days of primary admission, the additional data were also collected by reviewing the recorded files. The severity of stroke was assessed using the NIHSS score; as the score of 0 indicated no stroke symptoms, the score of 1–4 indicated minor stroke, the score of 5–15 indicated moderate stroke, the score of 16–20 indicated moderate-to-severe stroke, and the score of 21–42 indicated severe stroke. Using the baseline information, the level of risk for recurrent brain stroke was initially determined based on the ABCD2 scoring system. Then, by adding a new score for the presence of atrial fibrillation to ABCD2 system, the new scoring system as “ABCD2F” was introduced and the risk stratification was done again on this new system. The study endpoint was to determine the value of ABCD2 and ABCD2F systems to distinguish occurrence of the recurrent brain stroke within 90 days of primary event and also to determine the discriminative value across the two scoring systems.

Results were presented as mean ± standard deviation (SD) for quantitative variables and were summarized by absolute frequencies and percentages for categorical variables. Categorical variables were compared using Chi-square test or Fisher's exact test when more than 20% of cells with an expected count of less than 5 were observed. Quantitative variables were also compared with* t*-test or Mann-Whitney* U* test. A receiver operating characteristic (ROC) curve was used to determine the value of scoring systems to distinguish occurring from nonoccurring recurrent stroke and also to determine the sensitivity and specificity. For the statistical analysis, the statistical software SPSS version 16.0 for Windows (SPSS Inc., Chicago, IL) was used. *p* values of 0.05 or less were considered statistically significant.

## 3. Results

In total, 138 patients were included within the study with the mean age of 65.48 ± 12.03 years (ranged from 35 to 88 years) and 64.5% of them were males. Regarding the type of stroke, 76.8% had an ischemic stroke, and others suffered from TIAs. In electrocardiographic assessment, 64.5% had the normal pattern, while atrial fibrillation was revealed in 6.5%; furthermore, 13.0% had evidence of cardiac ischemic events, and 5.1% had cardiac blocks. With regard to initial symptoms on admission, 71.7% had weakness plus speech abnormality, 12.3% had speech abnormality alone, and 15.9% had symptoms related to posterior circulation. The duration of symptoms in patients who suffered from TIA was less than 10 minutes in 4.3%, while the symptoms lasted more than 60 minutes in 11.6% of them. In total, only 3.6% were admitted to ICUs on admission time. Regarding hemodynamic indices, 52.9% had first systolic blood pressure higher than 120 mmHg, 30.4% had first diastolic blood pressure higher than 90 mmHg, and 29.7% had first pulse rate higher than 90 beats per minute. Assessing cardiovascular risk factors showed the history of CVA in 13.0%, history of TIAs in 1.4%, history of ischemic heart disease in 24.6%, and history of valvular heart disease in 5.8%. Also, previous atrial fibrillation was found in 2.2%. Moreover, 28.3% were diabetic, 56.5% were hypertensive, 15.2% were hyperlipidemic, and 23.2% were smokers. With respect to oral medications, aspirin was used in 26.1%, clopidogrel in 2.9%, and warfarin in 5.8%. The mean ejection fraction of left ventricle was 50.46 ± 9.46 percent and, in 20.3% of cases, the ejection fraction of left ventricle was less than 35%. Left heart clot was only observed in 1.4% and left ventricle aneurism was observed in 4.3%, whereas wall motion abnormality in echocardiography was found in 18.8%. The mean of total initial NIHSS was 8.38 ± 5.26 and 1.06 ± 1.98 in patients with stroke and TIA, respectively. Based on NIHSS, in patients with ischemic stroke, 23.8% had mild stroke, 61.9% had the moderate stroke, 13.3% had moderate-to-severe stroke, and only 1.0% had severe stroke.

The follow-up of the patients for 90 days showed recurrent stroke, as TIAs or ischemic stroke was revealed in 9.4%.

No difference was found between the patients with and without 90-day clinical recurrent stroke in both ABCD2 score (4.62 ± 1.80 versus 5.06 ± 1.30; *p* = 0.406) and ABCD2F score (4.77 ± 1.96 versus 5.11 ± 1.31; *p* = 0.395). In total, a strong correlation was observed between the two scoring systems (*r* = 0.984; *p* < 0.001).

**Figure 1:**
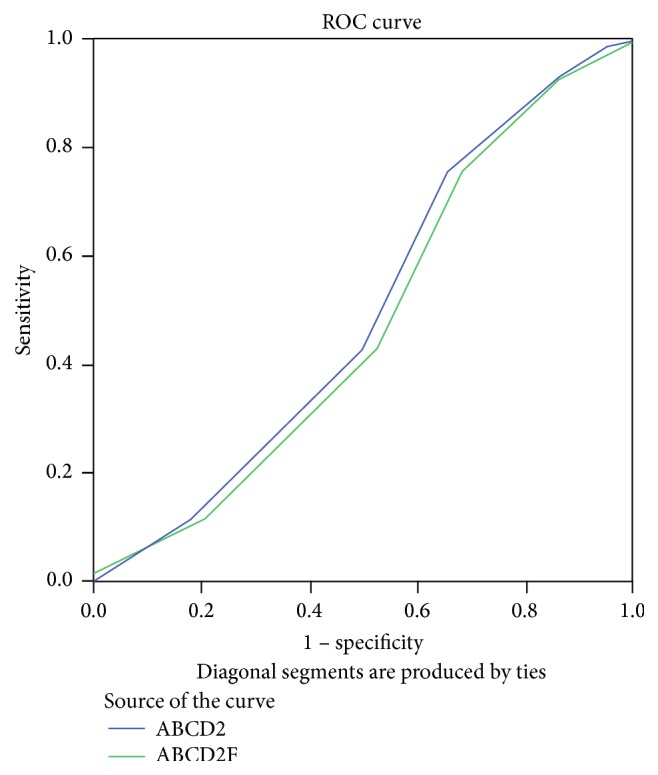
Area under the ROC curve analysis to assess the value of ABCD2 and ABCD2F tools to predict recurrent brain stroke.

Using the ROC curve analysis for determining the value of scoring systems to predict recurrent stroke, the area under the curve for ABCD2 was 0.434 (95% CI: 0.245–0.623; *p* = 0.433) and for ABCD2F it was 0.452 (95% CI: 0.256–0.648; *p* = 0.567), indicating low value of both systems for assessing recurrence of stroke within 90 days of the primary event (Figure 1).

Multivariable logistic regression analysis (Table 2) showed that none of the baseline factors could predict 90-day recurrent stroke.

## 4. Discussion

The current study attempted to assess ABCD2 scoring system of two aspects. First, we aimed to assess the value of this tool to predict occurring recurrent brain stroke within 90 days of first attacks. Second, we would like to examine whether adding occurrence of atrial fibrillation to this scoring system could enhance its value to predict recurrent brain stroke within this follow-up time. In this regard, the study had some important points. First, we showed that the ABCD2 scoring system was not valuable enough for predicting 3-month recurrent brain stroke. In other words, by using two statistical tools, including multivariable regression modeling and calculating area under the ROC curve, we could not demonstrate its high power to distinguish recurrent stroke. As previously noted, the ABCD2 scoring system was introduced to assess the risk for recurrent stroke during the first week of initial attack and thus its value to predict long-term stroke events had been already questioned. According to our results, it seems that the pure ABCD2 cannot be applied to assess mid-term or long-term recurrent cerebrovascular events, at least in our patients' population. Second, we also showed that even adding the parameter of atrial fibrillation could not improve the predicting value of ABCD2. In total, the low power of ABCD2 or its new version as ABCD2F can be explained by some reasons. First, it seems that, for improving its predictive value, we have to consider some other potential risk factors for delayed recurrent brain stroke admission etiologic stroke subtype, prior history of TIA/stroke, and topography, age, and distribution of brain infarcts. As also shown by Ay et al. [16], the new scoring system named RRE-90 including above parameters demonstrated good predictive performance with high reliability and also high external validity to predict 90-day risk of recurrent stroke. Thus, assessing this new scoring system among various populations such as our population is recommended. Second, a wide spectrum of risk factors has been identified as determinants for recurrent brain stroke. In other words, the causes of recurrent stroke are completely multifactorial. It has been indicated that diabetes and atrial fibrillation are two potential risk factors for mid-term recurrent attack of brain stroke [6]. Even the origin of stroke can be a major factor. In total, the relationship between risk factors such as ethnicity, diabetes mellitus, hypertension, and atrial fibrillation and stroke recurrence has been examined in several studies [3–6]. However, few had sufficient sample sizes or used multivariable analysis to control for confounding factors. Furthermore, no estimates have been made of the proportion of stroke recurrences that are attributable to these risk factors.

It should be noted that the risk factors for recurrence of stroke may be more complex and confounding. One of these major confounding factors is the type of the treatment following first cerebrovascular accident. From this point of view, the origin of stroke is very important and choosing of the type of treatment depends on it. As noted before, atrial fibrillation plays an important role in cardioembolic strokes. Also, Ois et al. showed that severe symptomatic cerebral arterial diseases have approximately five times risk for recurrence of stroke within 90 days [17]. The mode of the treatment in these conditions, including interventional procedures or medical treatments, affects the outcome of event, particularly recurrence of stroke. In multivariate analysis, surprisingly, we found that discharge with clopidogrel increases the risk for recurrent stroke by more than five times (OR = 5.23), but discharge with ASA and warfarin has OR of 0.69 and OR of 1.21, respectively. The possible explanation for these results can be the small number of patients who take clopidogrel (29 patients); however, a more detailed study is recommended to clarify this challenge.

In conclusion, it seems that either ABCD2 scoring system or ABCD2F scoring system cannot effectively predict 90-day recurrent brain stroke in our population. In other words, ABCD2 and/or atrial fibrillation are not good scoring candidates for assessing the risk of recurrent stroke within first 90 days. In this regard, it is better to examine the value of other confirmed scoring tools such as the RRE-90 system or to discover other potential risk profiles to structure the new scoring systems to predict recurrent brain stroke.

## 5. Conclusion

The ABCD2 and/or atrial fibrillation are not good scoring candidates for assessing the risk of recurrent stroke within first 90 days.

## Figures and Tables

**Table 1:** Inclusion and exclusion criteria of study.

Inclusion criteria	Exclusion criteria
(1) Patients with ischemic CVA (2) Patients with TIA (3) The complete presence of the data of the patient	(1) Patients with hemorrhagic stroke (2) Death within the admission period (3) Death within 90 days of discharge (4) Lack of follow-up within 90 days of discharge (5) Lack of enough follow-up data

**Table 2:** Multivariate logistic regression analysis to determine predictors of recurrent brain stroke.

Variable	*B*	Wald	*p* value	OR	95% CI for OR	
					Lower	Upper
Gender	−0.368	0.121	0.728	0.692	0.087	5.507
Age	0.033	0.835	0.361	1.034	0.962	1.111
Type of stroke	−0.128	0.054	0.815	0.880	0.300	2.579
NIHSS	−0.025	0.077	0.781	0.975	0.817	1.165
CVA	−0.746	0.301	0.583	0.474	0.033	6.805
IHD	0.559	0.342	0.559	1.749	0.269	11.397
DM	0.265	0.099	0.753	1.303	0.251	6.769
HTN	−0.469	0.314	0.575	0.625	0.121	3.233
HLP	−0.489	0.153	0.695	0.613	0.053	7.091
Smoking	−0.840	0.443	0.506	0.432	0.036	5.121
EF	−0.011	0.039	0.844	0.989	0.889	1.101
Discharge with ASA	−0.364	0.059	0.809	0.695	0.036	13.287
Discharge with clopidogrel	1.654	1.003	0.317	5.226	0.205	132.960
Discharge with warfarin	0.192	0.012	0.911	1.212	0.041	35.563
WMA	0.309	0.069	0.792	1.362	0.137	13.563
LV aneurysm	1.062	0.349	0.555	2.891	0.085	97.966
Constant	−3.577	0.501	0.479	0.028		

Hosmer-Lemeshow goodness of fit: *χ* ^2^ = 20.650; *p* = 0.008.

## References

[1] Beal C. C. (2010). Gender and stroke symptoms: a review of the current literature. *Journal of Neuroscience Nursing*.

[2] Rosamond W., Flegal K., Furie K. (2008). Heart disease and stroke statistics—2008 update: a report from the American Heart Association Statistics Committee and Stroke Statistics Subcommittee. *Circulation*.

[3] Petty G. W., Brown R. D., Whisnant J. P., Sicks J. D., O'Fallon W. M., Wiebers D. O. (1998). Survival and recurrence after first cerebral infarction: a population-based study in Rochester, Minnesota, 1975 through 1989. *Neurology*.

[4] Sacco R. L., Shi T., Zamanillo M. C., Kargman D. E. (1994). Predictors of mortality and recurrence after hospitalized cerebral infarction in an urban community: the Northern Manhattan Stroke Study. *Neurology*.

[5] Burn J., Dennis M., Bamford J., Sandercock P., Wade D., Warlow C. (1994). Long-term risk of recurrent stroke after a first-ever stroke. The Oxfordshire community stroke project. *Stroke*.

[6] Hillen T., Coshall C., Tilling K. (2003). Cause of stroke recurrence is multifactorial: patterns, risk factors, and outcomes of stroke recurrence in the South London Stroke Register. *Stroke*.

[7] Ghandehari K., Khajedaluei M. R., Yazdankhah Z., Ghandehari K. (2013). Risk factors of short-term stroke recurrence in patients with minor ischemic cerebrovascular events. *ARYA Atherosclerosis*.

[8] Elkind M. S. (2009). Outcomes after stroke: risk of recurrent ischemic stroke and other events. *The American Journal of Medicine*.

[9] Johnston S. C., Sidney S., Bernstein A. L., Gress D. R. (2003). A comparison of risk factors for recurrent TIA and stroke in patients diagnosed with TIA. *Neurology*.

[10] Galvin R., Geraghty C., Motterlini N., Dimitrov B. D., Fahey T. (2011). Prognostic value of the ABCD^2^ clinical prediction rule: a systematic review and meta-analysis. *Family Practice*.

[11] Wardlaw J. M., Brazzelli M., Chappell F. M. (2015). ABCD2 score and secondary stroke prevention: meta-analysis and effect per 1,000 patients triaged. *Neurology*.

[12] Giles M. F., Rothwell P. M. (2010). Systematic review and pooled analysis of published and unpublished validations of the ABCD and ABCD2 transient ischemic attack risk scores. *Stroke*.

[13] Guarino M., Rondelli F., Favaretto E. (2015). Short- and long-term stroke risk after urgent management of transient ischaemic attack: the Bologna TIA clinical pathway. *European Neurology*.

[14] Penado S., Cano M., Acha O., Hernández J. L., Riancho J. A. (2003). Atrial fibrillation as a risk factor for stroke recurrence. *American Journal of Medicine*.

[15] Kaarisalo M. M., Immonen-Räihä P., Marttila R. J. (1997). Atrial fibrillation in older stroke patients: association with recurrence and mortality after first ischemic stroke. *Journal of the American Geriatrics Society*.

[16] Ay H., Gungor L., Arsava E. M. (2010). A score to predict early risk of recurrence after ischemic stroke. *Neurology*.

[17] Ois A., Gomis M., Rodríguez-Campello A. (2008). Factors associated with a high risk of recurrence in patients with transient ischemic attack or minor stroke. *Stroke*.

